# Pediatric hemophagocytic lymphohistiocytosis: A rarely diagnosed entity in a developing country

**DOI:** 10.1186/s12887-021-02879-7

**Published:** 2021-09-18

**Authors:** Daniela Cleves, Viviana Lotero, Diego Medina, Paola M Perez, Jaime A Patiño, Laura Torres-Canchala, Manuela Olaya

**Affiliations:** 1 Pediatrics Department, Fundación Valle de Lili, Cali, Colombia; 2 Facultad de Ciencias de la Salud, Universidad Icesi, Cali, Colombia; 3 Pediatric Hematooncology Service, Fundación Valle de Lili, Cali, Colombia; 4 Primary Immunodeficiency (IDP) Group, Fundación Valle de Lili, Cali, Colombia; 5 Bone Marrow Trasplant Service, Fundación Valle de Lili, Cali, Colombia; 6 Pediatric Infectious Diseases Service, Fundación Valle de Lili, Cali, Colombia; 7 Centro de Investigaciones Clínicas, Fundación Valle de Lili, Cali, Colombia; 8 Pediatric Allergy-Immunology Service, Fundación Valle de Lili, Cali, Colombia

**Keywords:** Hemophagocytic, lymphohistiocytosis, pediatrics, Colombia, developing country

## Abstract

**Background:**

Hemophagocytic lymphohistiocytosis (HLH) is an exaggerated inflammatory reaction secondary to a host’s inadequate immune response causing a self-perpetuating loop of altered regulation. Signs and symptoms of HLH are compatible with other common diseases and are nonspecific. Underdiagnosis makes it difficult to estimate the real incidence of HLH, especially in developing countries.

**Methods:**

Retrospective, descriptive study of pediatric patients admitted to a high-complexity institution in Cali, Colombia between 2012 and 2019 with HLH diagnosis. Medical history review to complete an electronic database and a secondary, descriptive analysis was carried out. The study was approved by the Institutional Ethics Committee.

**Results:**

Twenty-one patients were included. 52.4 % of the population was male with a median age of 9.3 years [IQR (3.0-13.7 years)]. More than half of patients (66.6 %) had viral disease at diagnosis, the most frequent being Epstein-Barr Virus (EBV) (52.3 %) and dengue (14.3 %). Three patients had confirmed gene mutations (G6PC3, XIAP, and UNC13D). 95 % of the patients were treated with the HLH 2004 protocol, half of them received incomplete protocol with intravenous immunoglobulin (IVIG) and/or systemic steroids, while the other half received the complete protocol including etoposide and cyclosporine. More than three-fourths (76.2 %) required admission to an ICU with a median stay of 14 days [IQR (11–37 days)] and a median hospital stay of 30 days [IQR (18–93 days)]. 14.3 % (*n* = 3) of patients died.

**Conclusions:**

HLH is a complex disease that requires multidisciplinary management with secondary HLH due to EBV infection being a common cause. There is increasing awareness of HLH diagnosis in developing countries such as Colombia which can offer earlier treatment options and better outcomes.

## Background

Hemophagocytic lymphohistiocytosis (HLH) is an exaggerated and ineffective inflammatory reaction secondary to a host’s inadequate immune system response causing a self-perpetuating loop of altered immune system regulation.[1, 2] In HLH there is overactivation of T cells, natural killer (NK) cells and macrophages causing an uninhibited release of cytokines.[2, 3] The term hemophagocytosis describes the pathognomonic findings where highly activated macrophages taking up different cells including lymphocytes, erythrocytes, leukocytes, and platelets in different tissues, producing excessive cytokines and an uncontrolled inflammatory reaction.[1] The term HLH encompasses a wide range of disorders including primary HLH [which includes familial HLH (FHLH), familial erythrophagocytic lymphohistiocytosis] and secondary HLH [infection-associated hemophagocytic syndrome and autoimmune-associated macrophage activation syndrome (MAS)].[1, 3].

Signs and symptoms of HLH are nonspecific and usually compatible with other common diseases such as infections, tumors and rheumatological diseases.[4, 5] HLH should be included in the differential diagnosis of other clinical conditions such as: (1) fever of unknown origin, (2) hepatitis with coagulopathy (30 % of patients with HLH present an increase of transaminases above 100 U/L), (3) sepsis with multiple organic failure, (4) lymphocytic encephalitis.[6].

The first diagnostic guidelines for HLH were published by the Histiocyte Society in 1991 and included clinical, laboratory and histopathological criteria.[6, 7] In 2004 the guidelines were modified due to the fact that some patients did not fully complete all criteria and different criteria may develop throughout the course of the disease and as genetic testing has become more readily available.[6] Just as well, other criteria have been tested including the H-score.[8, 9].

Currently, underdiagnosis makes it difficult to estimate the real incidence of HLH. Some reports estimate 1.2 cases per million children per year.[1, 10, 11] These entities occur in children under 1 year of age in 70-80 % of cases and the incidence rate is 1 per 1 million newborns per-year.[5, 12–14] Some reports described a male predominance but there is not a clear association.[14, 15] To our knowledge, there are only two case reports in Colombia regarding HLH in pediatric patients, there are no reports of epidemiological trends in Colombia or in Latin America.[16, 17].

We aimed to determine the frequency of hemophagocytic syndrome and describe the demographic, clinical and outcome characteristics of pediatric patients who were hospitalized a high complexity institution in Cali, Colombia between 2012 and 2019.

## Methods

This is a retrospective, observational study evaluating patients younger than 18 years of age admitted to the pediatric emergency department (PED) in which HLH diagnosis was made between 2012 and 2019 in Fundación Valle de Lili (FVL), a high-complexity institution in Cali, Colombia. FVL is the pediatric reference center for complex pathologies in southwestern Colombia.

Data were obtained from the institutional statistics of patients under 18 years of age with ICD-10 (D76.1) diagnoses in electronic clinical records. Subsequently, a medical history review was carried out by a trained pediatrics resident to verify diagnosis and a database in BD Clinic, an institutionally created virtual platform for research data, was completed. The database included 120 variables including sociodemographic, clinical, laboratory, treatment/management and outcome variables.

Clinical and laboratory variables were included on diagnosis, 24–48 h afterwards, at 4 weeks and at 8 weeks. Not all patients included have data for 4 weeks and 8 weeks, because of adequate clinical response they were discharged from hospital. Most patients did not continue follow-up after discharge as follow-up consults are not authorized by health care providers in our health system hindering adequate long-term follow-up and management.

Diagnosis of HLH was based on the HLH-2004 Criteria:[6].

***With 5 out of 8 criteria the diagnosis is made***:

1. Fever.
2. Splenomegaly.
3. Cytopenias that affect at least 2 of 3 lineages in the peripheral blood and are not caused by hypocellular bone or marrow (neutrophils < 1 × 109/L, hemoglobin < 9 g/dL, platelets count < 100 × 10^9^/L).
4. Serum Triglycerides ≥ 3.0 mmol/L (≥ 265 mg/dl) or serum fibrinogen < 150 mg/dL.
5. Hemophagocytosis in bone marrow (BM), spleen, or lymph nodes with no evidence of malignancy.
6. Ferritin ≥ 500mcg/L.
7. Soluble CD 25 ≥ 2,400 U/m (*not available in our institution).
8. Reduced or absent NK cell activity.

**OR**

A molecular or genetic test confirming the presence of primary HLH.

Genetic testing in our institution is not readily available and is only indicated in certain cases when Genetics considers it pertinent. In young patients with HLH criteria or refractory cases of HLH, whole exome sequencing or FHLH NSG panel are done.

Our institutional protocol states that once diagnosis of HLH is suspected based on clinical and laboratory parameters, a stepwise treatment approach is initiated. The first step includes administration of intravenous immunoglobulin (IVIG) during 3 doses. If there is no improvement in inflammatory marker values or clinical signs and symptoms within the first 24 h, steroids (dexamethasone) are then started. Reevaluation takes place continuously, if there is no improvement within 24–48 h after initiating steroids, then chemotherapy with cyclosporine and etoposide is added. Intrathecal methotrexate is added to management if there is evidence of neurological compromise. Patients usually receive 3 doses of IVIG and two weeks of steroids if there is clinical improvement and don’t require chemotherapy. Patients that receive complete HLH-04 protocol complete 8 weeks of chemotherapy with cyclosporine, etoposide, and dexamethasone in addition to the 3 doses of IVIG. Patients that only receive IVIG or IVIG and steroid are considered to receive incomplete or partial HLH-04 protocol. If after 6 weeks of treatment, patients continue to show little or no improvement in inflammatory markers and HLH-04 criteria, compatibility studies for bone marrow transplant (BMT) are started.

With the data, a descriptive and secondary analysis of the data was carried out using STATA 12.1 ©. Frequency, central tendency and dispersion measures were used according to the classification of each of the variables and their non-normal distribution.

The study was approved by Fundación Valle de Lili’s Institutional Ethics Committee (#1273).

## Results

A total of 21 pediatric patients between 2012 and 2019 were diagnosed with some form of HLH (either primary or secondary) in our institution. Males were 52.4 % of the population with a median age of 9.3 years [range (3.0-13.7 years)]. The majority of patients were from Cali (the city where FVL is located) (38.1 %); however, we also had patients from other parts of the Valle del Cauca state (23.8 %), Cauca department (19 %), Nariño state (9.5 %), Choco state (4.8 %) and Caquetá state (4.8 %). All patients were febrile on admission with a median of 17 days (IQR 6–30 days) while 1/3 of patients had splenomegaly (Table 1).

**Table.1:** Diagnostic criteria at admission in patients between 0 and 18 years with a diagnosis of HLH in the FVL between 2012 and 2019 (n = 21)

**Criteria at admission**	**Frequency** **%(n)**	**Median (IQR)**
Fever ≥ 7 days	71.4(15)	17 days(6–30)
Splenomegaly > 3 cm below rib limit	61.9(13)	-
Bicytopenia	61.9(13)	-
Hyperferritinemia > 500 mg/dl	81.0(17)	1866.5 ng/ml (889-10286)
Hypertriglyceridemia (> 150 mg/dl) or hypofibrinogenemia (< 150 mg/dl)	76.2(16)	267 mg/dl (184–324)
		167.5 mg/dl (94–315)
Hemophagocytosis in BM	57.1(12)	-
Decrease in NK cells or altered functionality	14.3(3)	-
No evidence of malignancy	100.0(21)	-
Genetic Testing for primary HLH^a^	14.3(3)	-

^a^Genetic testing is not widely available and is only performed in select cases

**In our institution we have no availability of soluble CD 25

Additionally, on diagnosis the most common blood cell alterations were anemia and thrombocytopenia.

Two patients (9.5 %) had bacterial growth in cultures, one patient with abdominal *Mycobacterium tuberculosis* and another patient with *Pseudomona aeruginosa* and *Klebsiella pneumoniae* in blood. Fourteen patients (66.6 %) had viral isolation, with EBV (11 patients, 52.3 %) and dengue (3 patients) being most frequent viral etiologies.

Genetic testing was performed in 3 individuals. Two patients were tested via whole exome sequencing. The first patient had two variants: *G6PC3* (NM_138387.3): c.1 A > G, p.Met1Val, heterozygous, classified as pathogenic, and *LYST* (NM_000061.3): c.7786 C > A, p.(Arg2596=), heterozygous, classified as likely benign.

The second patient showed this variant: *XIAP* (NM_001167.3): c.1045G > T, p.Glu349Ter, hemizygous, classified as pathogenic. The remaining patient was tested via multigene panel for familial hemophagocytic lymphohistiocytosis, which revealed this variant: *UNC13D* (NM_199242.3): c.3049G > A, p.Glu1017Lys, homozygous, classified as likely pathogenic.

**Fig. 1:**
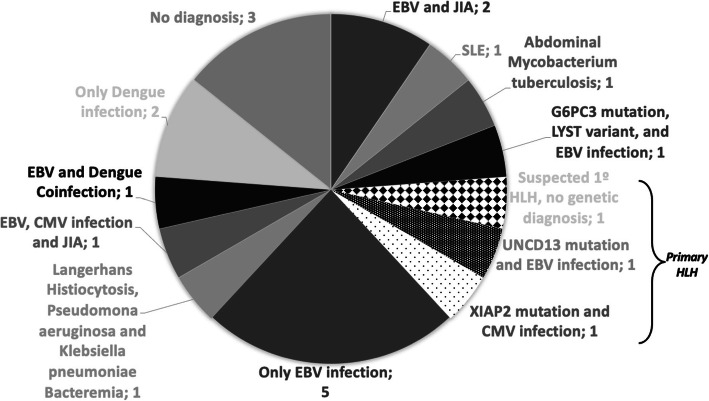
Different etiologies and triggers of patients between 0 and 18 years of age diagnosed with HLH in the FVL between 2012 and 2019

The patients with the G6PC3 and UNC13D mutations had acute EBV infection and the patient with the XIAP mutation had acute cytomegalovirus (CMV) infection, triggering the HLH. The patients with XIAP and UNC13 mutations underwent BMT, while the patient with G6PC3 mutation had a heterozyte form and responded adequately to IVIG management after an EBV infection. No partial albinism was evident in the patient with the LYST variant. (Fig. 1)

Most patients received management with HLH 2004 protocol (95.2 %), 47.6 % received incomplete protocol (only human intravenous immunoglobulin and/or dexamethasone at high doses) while the other half of the patients received the complete protocol (etoposide, cyclosporine and dexamethasone at high doses). One patient received methotrexate at another institution before referral to our institution and underwent BMT. (Table 2)

**Table.2:** Treatment and outcome of patients between 0 and 18 years of age diagnosed with HLH in FVL between 2012 and 2019

	Cause HLH	Age at diagnosis (years)	Only IVIG	Dexametasone and IVIG	Dexametasone, IVIG, etoposide and cyclosporine	BMT	Systemic Compromise	Alive
**1**	FHLH (LYST mutation) EBV infection	5			X	No	No	Yes
**2**	FHLH (UNCD13 mutation) EBV infection	3			X	Yes	Acute liver failure, CNS compromise	Yes
**3**	FHLH (XIAP mutation) CMV infection	0.17			X	Yes	Severe dehydration secondary to diarrhea	Yes
**4**	Suspected FHLH, no genetic confirmation	0.75			X	Yes	CNS compromise	Yes
**5**	EBV infection (IgM)	3			X	No	No	Yes
**6**	EBV infection (Persistently elevated viral load)	13			X^a^	No	Respiratory and renal failure	No
**7**	EBV infection (Elevated initial viral load)^b^	13				No	No	Yes
**8**	EBV infection (Elevadted viral load)	8	X			No	No	Yes
**9**	EBV infection (Persistently Elevated viral load)	13			X	No	Respiratory failure	No
**10**	EBV Infection (elevated viral load) + JIA diagnosis	15	X			No	No	Yes
**11**	EBV Infection (biopsy of ganglion) + JIA diagnosis	9			X	Yes	No	Yes
**12**	Dengue (positive IgM) + EBV (Elevated viral load)	11	X			No	No	Yes
**13**	Dengue (positive IgM)	0.33	X			No	No	Yes
**14**	Dengue (positive IgM)	11	X			No	No	Yes
**15**	CMV (positive viral load) + EBV (Positive viral load) + JIA diagnosis	6		X		No	No	Yes
**16**	Abdominal Mycobacterium tuberculosis infection	15		X		No	Respiratory, renal and hepatic failure	Yes
**17**	SLE	14		X		No	No	Yes
**18**	Langerhans Histiocytosis + Pseudomona aeruginosa and Klebsiella pneumoniae Bacteremia	1	X			No	No	Yes
**19**	Suspected Autoimmunity, diagnosis pending	11		X		No	No	Yes
**20**	Unknown	1			X^c^	No	Respiratory, renal and hepatic failure	No
**21**	Unknown	16	X			No	No	Yes

^a^ Died 4 weeks after starting chemotherapy

^b^ Self-limited course of disease, follow-up for suspected rheumatological disease

^c^ Died 3 weeks after starting chemotheraphy

FHLH: Familial Hemophagocytic Lymphohistiocytosis. CMV: Cytomegalovirus. EBV: Ebstein Barr Virus. JIA: Juvenile Idiopathic Arthritis. SLE: Systemic Lupus Erythematosus. IVIG: Intravenous Immunoglobulin. CNS:Central Nervous Systema

A total of 3 patients underwent BMT, two with genetic confirmation for FHLH (UNCD13 and XIAP mutations), and another patient with suspected FHLH due to age of debut (2 months) but pending genetic confirmation. All 3 patients were under 5 years of age and underwent allogenic BMT with haploidentical donor. The donors were family members chosen according to the institutional preferences of donor selection. The conditioning regimen used was reduced intensity conditioning (RIC) based on fludarabine and low-dose cyclophosphamide with the addition of busulfan, all patients were alive one year after BMT with complete immune reconstitution.

More than 76 % of patients (76.2 %) required admission to an intensive care unit with a median stay of 14 days [IQR (11–37 days)] and a hospital stay of 30 days [IQR (18–93 days)]. Three patients (14.3 %) died before genetic testing could confirm final diagnosis, two of them had elevated EBV viral loads with active infection, one died before completing 4 weeks of HLH- 2004 protocol. The third patient did not have any infectious etiology, a rheumatological disease was suspected, however, he had a fulminant course of the disease and died two weeks after diagnosis was made.

**Fig. 2:**
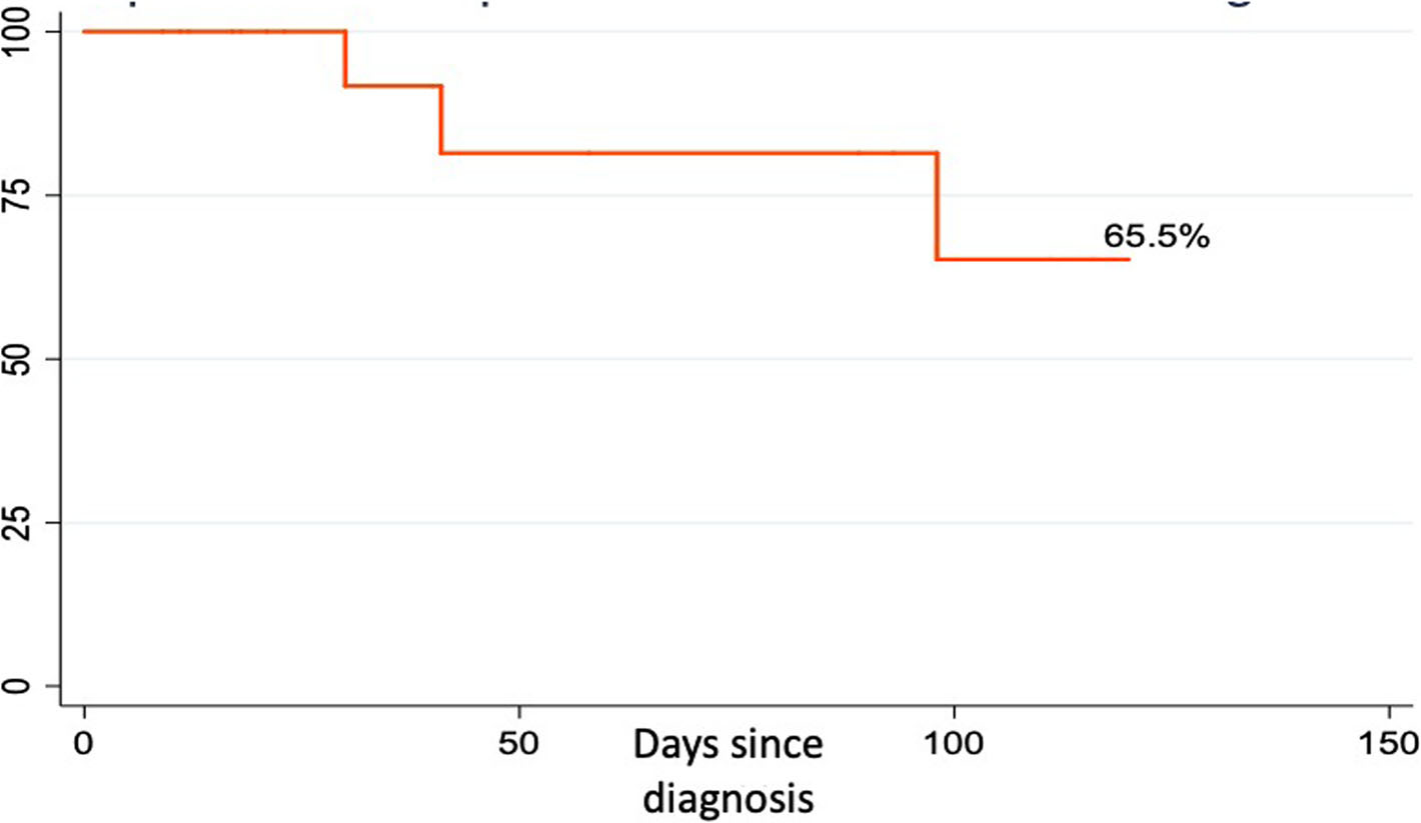
Survival of patients between 0 and 18 years of age diagnosed with HLH e in the FVL between 2012 and 2019

Additionally, overall survival rate of pediatric patients with HLH diagnosis was 65.5 % at a hundred days after diagnosis. (Fig. 2)

## Discussion

HLH is an underdiagnosed pathology especially in developing countries where lack of resources and awareness of this disease prevails. This is quite evident when we take into account the median duration of fever before diagnosis [17 days (IQR 6–30)], added to the fact that most of the patients were referred to our institution from centers with lower levels of complexity with a suspicion of malignancy and/or oncologic disease. Awareness of this pathology has increased in our institution where diagnosis has increased over the last couple of years because of an active search for the disease in critically ill patients and a new Pediatric Immunology Service.

HLH has a broad spectrum of etiologies, including infections which have a wide range of clinical presentations with high morbidity and mortality rates secondary to the host’s immune predisposition.[1, 3, 18, 19] Acute infection with EBV was the most frequent infection in our population, which is in accordance with what’s reported in literature.[18, 20] Dengue was the second most common virus associated with HLH we consider that it may be secondary to an active search of these infections due to our local epidemiology, and this is in accordance to literature in developing countries.[21] Serotype differentiation is not done in our institution.

Most of our patients had hypertriglyceridemia, hypofibrinogenemia and hyperferritinemia. Upon admission, 28 % of our population had ferritin > 10,000 ng/ml which has reported a sensitivity and specificity above 80 %.[22, 23].

Hemophagocytosis was found in bone marrow (BM) in 57 % of patients which is in accordance with literature reports depending on the time of the BM aspirate, this finding is reported between 40 and 80 %.[2, 24] In our population hemophagocytosis was not sought in tissues other than BM since this practice is not protocolized in our institution.

The rapid onset of immunosuppressive therapy to control host immune hyperreactivity is necessary to obtain better results.[1–3, 6, 25] The first-line treatment is dexamethasone based on its cytotoxic effect on lymphocytes, inhibition of cytokine production and dendritic cell. [1–3, 6] Additionally, it can be combined with cyclosporine A, which interferes with the activation of lymphocyte and macrophage function. ^1–3,6^ The etoposide is used as it destroys the antigen presenting cells. [1–3, 6] The HLH-94 trial, published in 2011, was the largest prospective diagnostic/therapeutic study of HLH before de HLH 04 report.[26] They evaluated the results of an 8-week course of treatment with dexamethasone, cyclosporine and etoposide and reported a reduction in the mortality rate from 95 to 30 %.[26] While this study was being conducted, the HLH-2004 protocol was initiated with revised diagnostic criteria to achieve better remission by adding cyclosporine to the treatment and intensifying the induction regimen. [26] In our patients, 42.5 % received a complete protocol with cyclosporin A and etoposide, of which 2 died before reaching 8 weeks of treatment and without genetic studies to clarify the origin of HLH, while three patients underwent BMT.

Three of our patients underwent BMT, 2 with genetic confirmation of FHLH (UNCD13 and XIAP mutations), while the third patient underwent BMT without confirmed genetic mutation but with a highly suspicious clinical picture as the patient was less than one-year old with persistence of HLH symptoms after 8 weeks of treatment in the HLH-2004 protocol. All three patients evolved adequately after BMT with 100 % survival and little complications one year after BMT. Patients with genetic causes, persistent/resistant HLH treatment or reactivation of the disease should have a BMT as soon as possible to improve their survival and reduce morbidity.[6].

It is worth mentioning that genetic testing is done in specific cases, usually in patients under five years of age where primary HLH would be most likely. Genetic testing in our country is not readily available. Four years ago, national legislation was approved to include universal coverage of genetic testing in our health system allowing greater access to these studies; however, there is still a prolonged process before the authorization to test individual patients that hinder fast results.[27] Additionally, elevated costs and limited availability of centers that have adequate equipment and methods for genetic testing delays accessible results.

Most of our patients under 5 years of age required chemotherapy, which indicates more severe compromise secondary to HLH and is possibly related to an underlying genetic alteration. Two of our patients over the age of 5 years that required chemotherapy had more severe disease and both died before completing treatment with chemotherapy, both had severe and persistent EBV infection. All other patients over the age of 5 years responded well to treatment with IVIG, alone or in combination with dexamethasone. In any case we must do laboratory tests every 24 h initially, as HLH is a rapidly progressing disease, and at the slightest clinical or laboratory deterioration we must advance in treatment rapidly to ensure better results in a stepwise approach as previously mentioned.

Patients with HLH resistant to etoposide-based therapy have a poor prognosis because there are currently few treatment alternatives with little evidence available. Alemtuzumab, a monoclonal antibody directed to the CD52 antigen in lymphocytes, monocytes, macrophages, and dendritic cells has been proposed for refractory HLH. [28, 29] Just as well, rituximab has recently been proposed as an alternative treatment for EBV-associated HLH.[28, 29] Several reports have also reported good outcomes after use of ruxolitinib in refractory or relapsed HLH.[30, 31].

With regards to FHLH, Locatelli et al. recently published findings on the efficacy and safety of emapalumab, a human anti–interferon-γ antibody, in combination with dexamethasone in 34 pediatric patients with primary HLH with good outcomes and no toxicity.[32].

The Hybrid Immunotherapy for Hemophagocytic Lymphohistiocytosis (HIT-LHL) study is an open label phase II trial that was carried out by Cincinnati group in patients with HLH < 18 years of age.[33] They evaluated the safety and effectiveness of a treatment regimen combining ATG with dexamethasone, intrathecal methotrexate, etoposide and hydrocortisone.[33] The primary outcome was complete response rate at 8 weeks (clinicaltrials.gov NCT01104025).[33] Euro-HIT-LIT is a phase II/III trial and a Cincinnati study extension performed in Europe (ClinicalRegister.eu 2011-002052-14).[34] There still no result of both trials, but preliminary data is promising on the effectiveness of treating HLH with hybrid immunotherapy. [33, 34]

Some of the limitations with our study includes its retrospective nature over 7 years, which makes it difficult to gather some of the data that wasn’t recorded is medical records and could include information or selection bias in our data. We also consider the loss of follow-up after discharge to be a major limitation, however, this is common in our health system which is plagued by bureaucratic processes making it sometimes difficult for patients to access specialty care.

## Conclusions

Early inclusion of HLH in differential diagnoses could reduce its insufficient and late diagnosis and improve patient outcomes. Diagnosis can be made with clinical and paraclinical criteria while delays in genetic testing should not delay treatment. New therapies are emerging to improve the prognosis and improve the quality of life of patients with HLH.

## Data Availability

The data that support the findings of this study are available on request from the corresponding author, Dr Manuela Olaya. The data are not publicly available due to the international ethical regulations that protect the data security of the subjects.

## Abbreviations

HLH: Hemophagocytic lymphohistiocytosis
EBV: Epstein-Barr Virus
FHLH: Familial hemophagocytic lymphohistiocytosis
NK: Natural killer
MAS: macrophage activation syndrome
FVL: Fundación Valle de Lili 7
CMV: Cytomegalovirus
BMT: bone marrow transplant
BM: Bone Marrow
LDH: Lactic Deshydrogenase
JIA: Juvenile Idiopathic Arthritis
SLE: Systemic Lupus Erythematosus
IVIG: Intravenous Immunoglobulin
CNS: Central Nervous System
